# Characterization of Porcine Aortic Valvular Interstitial Cell ‘Calcified’ Nodules

**DOI:** 10.1371/journal.pone.0048154

**Published:** 2012-10-26

**Authors:** Kristy L. Cloyd, Ismail El-Hamamsy, Suwimon Boonrungsiman, Martin Hedegaard, Eileen Gentleman, Padmini Sarathchandra, Francesca Colazzo, Molly M. Gentleman, Magdi H. Yacoub, Adrian H. Chester, Molly M. Stevens

**Affiliations:** 1 Department of Materials, Imperial College London, London, United Kingdom; 2 Institute of Biomedical Engineering, Imperial College London, London, United Kingdom; 3 Department of Bioengineering, Imperial College London, London, United Kingdom; 4 Harefield Heart Science Centre, Imperial College London, Harefield, Middlesex, United Kingdom; 5 Division of Cardiac Surgery, Montreal Heart Institute, Montreal, Canada; 6 Department of Mechanical Engineering, Texas A&M University, College Station, Texas, United States of America; Brigham and Women's Hospital, Harvard Medical School, United States of America

## Abstract

Valve interstitial cells populate aortic valve cusps and have been implicated in aortic valve calcification. Here we investigate a common *in vitro* model for aortic valve calcification by characterizing nodule formation in porcine aortic valve interstitial cells (PAVICs) cultured in osteogenic (OST) medium supplemented with transforming growth factor beta 1 (TGF-β1). Using a combination of materials science and biological techniques, we investigate the relevance of PAVICs nodules in modeling the mineralised material produced in calcified aortic valve disease. PAVICs were grown in OST medium supplemented with TGF-β1 (OST+TGF-β1) or basal (CTL) medium for up to 21 days. Murine calvarial osteoblasts (MOBs) were grown in OST medium for 28 days as a known mineralizing model for comparison. PAVICs grown in OST+TGF-β1 produced nodular structures staining positive for calcium content; however, micro-Raman spectroscopy allowed live, noninvasive imaging that showed an absence of mineralized material, which was readily identified in nodules formed by MOBs and has been identified in human valves. Gene expression analysis, immunostaining, and transmission electron microscopy imaging revealed that PAVICs grown in OST+TGF-β1 medium produced abundant extracellular matrix via the upregulation of the gene for Type I Collagen. PAVICs, nevertheless, did not appear to further transdifferentiate to osteoblasts. Our results demonstrate that **‘**calcified’ nodules formed from PAVICs grown in OST+TGF-β1 medium do not mineralize after 21 days in culture, but rather they express a myofibroblast-like phenotype and produce a collagen-rich extracellular matrix. This study clarifies further the role of PAVICs as a model of calcification of the human aortic valve.

## Introduction

The aortic valve performs a number of sophisticated functions including regulation of unidirectional oxygenated blood flow from the heart to the rest of the body [1], [2]. These critical functions are dependent on the unique structure of the valve at the tissue, cellular and molecular levels [3]. Aortic valve calcification, a disruption to the intricate structure of the valve through the accumulation of mineral deposits in the valve tissue cusps, leads to considerable morbidity and mortality. The disease increases in prevalence with age [4], and will continue to increase as the world’s population ages [5]. Currently, there is no proven pharmacotherapy to prevent or limit aortic valve calcification progression. The treatment of choice for aortic valve calcification, therefore, is to surgically replace valves with bioprosthetic or mechanical alternatives [6]–[8] and is associated with many complications including progressive calcification of the replacement valve. Pharmacotherapies that prevent native and prosthetic valve calcification remain elusive, partially due to a lack of understanding of the pathophysiological mechanisms that regulate disease progression and the lack of a proven *in vitro* disease model [3], [9], [10].

Valvular Interstitial Cells (VICs) are the most abundant cell type in the aortic valve and play a vital role in maintaining valve function [11]. VICs represent a heterogeneous population of cells comprised of embryonic progenitor, endothelial/mesenchymal, progenitor, quiescent (fibroblasts), activated (myofibroblasts) and osteoblastic phenotypes [11]–[14], which are thought to play a role, either alone or collectively, in the process of calcification [12], [15]. Several studies have suggested that VICs may transdifferentiate to bone-forming cells and directly mediate the formation of calcified mineral deposits, in a process analogous to bone formation [4], [6], [16]. Additionally VICs have been implicated in a dystrophic calcification through VICs activation in combination with apoptotic events resulting in calcium salt deposition [17].

VICs derived from porcine tissue (PAVICs) are often used as a model for aortic valve research, as the fast-growing and readily available cells can be cultured *in vitro* [18]. They have been reported to be a particularly valuable model for aortic valve calcification studies, as distinct cellular nodules spontaneously form in culture in the presence of control medium and osteogenic medium supplemented with transforming growth factor beta 1 (TGF-β1) [17], [18]. TGF-β1 has been implicated in the pathobiology of aortic valve stenosis and has been demonstrated to co-localize with calcification in diseased aortic tissue cusps [17], [19]. Cellular nodules formed in the presence of TGF-β1 are often referred to in the literature as ‘calcifying nodules’ [17], [20]. Nevertheless, a relationship between *in vitro* nodule formation and pathological aortic valve calcification has not been clearly established, and the cellular composition and nature of the material that comprises such nodules has yet to be determined. The hard material that accumulates on human calcified valves has been identified as a mixture of calcium phosphate phases [21]–[23], thus similar mineral species should be identifiable in a cell-mediated model of aortic valve calcification. The calcified material found in diseased human valves has been suggested to be a result of an osteoblast like mineralization and/or via a dystrophic calcification mechanism [16], [24].

Here we investigated nodule formation and composition in cultured PAVICs grown in osteogenic (OST) medium supplemented with TGF-β1 (OST+ TGF-β1) by carrying out a biological and materials-based characterization of the extracellular matrix (ECM) produced. We then compared this material to nodules formed by mineralizing murine calvarial osteoblasts (MOBs) and to PAVICs maintained in basal (CTL) medium. We utilized a combination of gene expression, immunohistochemistry, histochemical staining, scanning electron microscopy (SEM), transmission electron microscopy (TEM), and live cell bio-molecular analysis with micro-Raman spectroscopy, a rapid, noninvasive technique based on light scattering which reveals the molecular fingerprint of a sample without dyes or labels. Micro-Raman spectroscopy additionally enables the visualization of calcium salt deposition more specifically than traditionally used Alizarin Red S and will detect the presence of any calcium phosphate inclusion within the PAVICs nodules regardless of an osteoblast-like or dystrophic deposition [25], [26]. Our results suggest that PAVIC nodules grown in OST+TGF-β1 for up to 21 days show no evidence of calcium phosphate formation and show no indication of undergoing an osteoblastic differentiation, however, they do produce an abundant ECM which is predominately collagen including type I collagen.

## Materials and Methods

This study was reviewed and approved by the North London Research Ethics Committee (reference # 10/H0724/18), registered with the Royal Brompton & Harefield NHS Foundation Trust and performed in accordance with the requirements of the research governance framework. Human calcified aortic valve collection and isolation is fully described in Methods S1.

### VICs Isolation

Whole hearts from 18 to 24 month old pigs were obtained from an abattoir (Cheale Meats, Essex, U.K.). Aortic valve cusps were removed within 12 hours of slaughter and PAVICs were isolated through two collagenase digestions as previously described [18].

### Cell Culture

PAVICs were cultured in CTL medium consisting of high glucose Dulbecco’s Modified Eagles Medium (DMEM) supplemented with 1% (v/v) antibiotic–antimycotic, 10% (v/v) fetal bovine serum (FBS), and 2 mM L-Glutamine (All Invitrogen). OST+TGF-β1 medium consisted of CTL medium supplemented with 10 mM β-glycerophosphate, 10^−7^ M dexamethasone, 10^−6^ M ascorbic acid, and 10 ng/mL TGF-β1 (all Sigma-Aldrich). Media was replenished every three days. CTL+TGF-β1 medium consisted of CTL medium supplemented with 10 ng/mL TGF-β1. OST medium consisted of CTL medium supplemented with 10 mM β-glycerophosphate, 10^−7^ M dexamethasone, and 10^−6^ M ascorbic acid. MgF_2_ coverslips (micro-Raman spectroscopy), glass cover slips (histology) and tissue culture plastic were seeded with 5×10^4^ cells/cm^2^ at passages four or five. Prior to cell seeding, MgF_2_ and glass cover slips were incubated in FBS for 6 hours.

MOBs were enzymatically derived from the calvaria of neonatal mouse pups as previously described [27]. To form mineralized nodules, cells were cultured in alpha minimum essential medium (Invitrogen) supplemented with 15% (v/v) FBS, 2 mM L-glutamine, 10 mM β-glycerophosphate, 50 µg/ml ascorbic acid and 10^−6^ M dexamethasone from day 14. All MOBs were at passages two to four during testing.

### PAVICs Nodule Cross Section Preparation for Histology

After 7, 14 and 21 days, cultures were fixed in 4% (w/v) formaldehyde (FA) for 20 minutes at room temperature and then rinsed in phosphate buffered saline (PBS). For histological sectioning, cells were carefully scraped from the culture surface using a rubber policeman and re-suspended in a 1% (w/v) agarose (Sigma) in PBS using the method described by Gruber *et al*. [28]. Agarose gels containing VICs were again fixed in 4% (w/v) FA for 1 hour, dehydrated in a graded ethanol series and embedded in paraffin. 5 µm sections were collected on glass slides and prepared for staining.

### Modified Verhoeff van Geison for Detection of Collagen, Elastin, Muscle and Cell Nuclei

Samples were stained using an elastin stain kit (Sigma) which utilizes the modified Verhoeff van Gieson method to stain elastic fibres blue-black to black, collagen pink to red, muscle yellow and nuclei blue to purple. Sections from blood vessels of adult mice were used for a positive control.

### Immunoperoxidase for Detection of Smooth Muscle Alpha Actin (αSMA)

Prior to immunoperoxidase staining, sections were dewaxed, rehydrated in nanopure distilled water (dH2O) and washed in PBS for 5 minutes. Slides were immersed in 0.1 M citrate buffer (pH 6) and microwaved for 10 minutes before being immersed in peroxide (0.01% w/v) in PBS for 10 minutes. Sections were then washed 3 times for 5 minutes each in PBS and blocked with 3% (w/v) bovine serum albumin (BSA) in PBS for 30 minutes. Sections were incubated separately for 1 hour with primary antibodies (Sigma).

Sections were incubated with biotinylated goat anti-mouse immunoglobulins (GAM IgG-Vector laboratories) for 1 hour, washed 3 times in PBS and then incubated for 1 hour with Avidin-Biotin Complex (ABC-Vector laboratories). Reactivity was detected using diaminobenzidine tetrahydrochloride (DAB tablets- Sigma) (25 mg/ml) and hydrogen peroxide (0.01% w/v). Sections were then counter stained with haematoxylin.

### Immunostaining for αSMA and DNA

Sections were dewaxed and rehydrated in dH20. Antigen retrieval was carried out by immersing slides in 0.1 M citrate buffer (pH 6), placing in a microwave for 10 minutes, incubating in citrate buffer for a further 20 minutes, and rinsing with tap water. To reduce non-specific binding, slides were incubated with 3% (w/v) BSA for 30 minutes. Specimens were then incubated with a smooth muscle alpha actin (αSMA) antibody (DAKO) for 1 hour at room temperature. Negative controls were incubated with 3% (w/v) BSA in PBS. After thorough washing, sections were incubated with goat anti mouse (IgG) Alexa Fluor 594 (Invitrogen) for 1 hour. After washing twice with PBS, cells were stained with DAPI and specimens mounted using Permafluor (Beckman Coulter).

### Alizarin Red S Staining for Calcium Detection

Cultures were fixed in 2% (v/v) FA for 10 minutes, washed in dH20 and stained for 10 min in 2% (w/v) Alizarin Red S (ARS) (Sigma) in dH2O, rinsed again, air dried, mounted on glass slides using DPX mounting medium and viewed using bright field microscopy.

### Gene Expression Analysis for Collagen I and BGLAP

Gene expression analysis was performed after 7, 14 and 21 days in culture. RNA was extracted using the QIAGEN Mini extraction kit according to the manufacturer’s instructions. Reverse transcription (RT) and real-time polymerase chain reaction (PCR) was performed as described [20]. TaqMan assays were purchased for type 1 collagen (*COL1A1*) and bone gamma-carboxyglutamate protein (*BGLAP*) (Ss03373340_m1, Ss03373655_s1) respectively; (Applied Biosystems). Target gene data were normalized against 18S ribosomal RNA levels (Cat. No. 4310893E; Applied Biosystems) and analyzed using the comparative cycle threshold (Ct) method.

### Scanning Electron Microscopy (SEM)

Cultures were fixed in 4% (v/v) FA in PBS for 45 minutes at 4°C and dehydrated in a graded ethanol series, followed by critical-point drying with hexamethyldisilazane (Sigma). Samples were sputter-coated with gold and viewed using a Leo 1525 Gemini scanning electron microscope with an EDX detector (Carl Zeiss SMT Ltd.) operated at 15 kV.

### Transmission Electron Microscopy (TEM)

Cultures were fixed in 4% (v/v) glutaraldehyde in 0.1 M PIPES buffer (pH 7.4) at 4°C for 2 hours and then fixed in 1% (w/v) osmium tetroxide in 0.1 M PIPES buffer at room temperature for 1 hour. The samples were then dehydrated using a graded ethanol series from 50%, 70%, 90% and 100% (v/v), followed by immersion in acetronitrile. The nodules were then progressively infiltrated with a Quetol based resin (12.6 g Quetol, 15.5 g Nonenyl succinic anhydride (NSA), 6.5 g Methyl nadic anhydride (MNA) and 0.6 g Benzyl dimethylamine (BDMA)) with ratio of 1∶1, 3∶1 (resin: acetonitrile) and pure resin for 2 hours, overnight and 4 days, respectively. Pure resin was changed every 24 hours. Embedded samples were polymerized at 60°C for 24 hours. 70 nm thick sections were cut onto a water bath via ultramicrotomy. The sections were collected immediately on bare 300 mesh copper TEM grids; selected sections were post-stained with uranyl acetate and lead citrate. TEM was performed on the Joel 2000 operated at 120 kV.

### Raman Spectroscopy

Live cell spectra were collected with a 785 nm laser, using a Renishaw InVia spectrometer connected to a Leica microscope as previously described [29]. Briefly, spectra were collected from live cell cultures maintained at 37°C in PBS supplemented with Mg and Ca. Spectra were collected over 5 accumulations of 3 second scans covering the Raman shifts range of 800–1800 cm^−1^. Samples were kept outside the incubator during testing for no longer than 30 minutes.

Raman spectra were pre-processed for background removal (baseline subtraction using weighted least squares) and multiplicative scattering correction [30], [31]. Interval partial least squares discriminate analysis (iPLS-DA) was applied to determine if a model could distinguish between treatment groups [32]. This model was derived using 194 total spectra collected from PAVICs nodules grown for 21 days in CTL or OST+TGF-β1 media.

For mapping of PAVICs nodules, cultures were fixed in 4% (v/v) FA in PBS for 45 minutes at 4°C and dehydrated in a graded ethanol series. Raman spectra were collected using a 532 nm laser, on a Renishaw InVia spectrometer connected to a Leica microscope. Spectra were collected using 1 accumulation of 10 seconds covering the Raman shifts range of 670–1500 cm^−1^.

Raman spectroscopy performed on diseased human aortic valve tissue is fully described in Methods S1.

### Statistical Methods

All continuous data are presented as mean ± standard deviation. For RT-PCR, all data were compared using the Mann-Whitney test for statistical significance. *p*-values <0.05 were considered significant.

## Results

### PAVICs Grown in OST+TGF-β1 Media form Nodular Structures and Stain Positive for ARS

**Figure 1:**
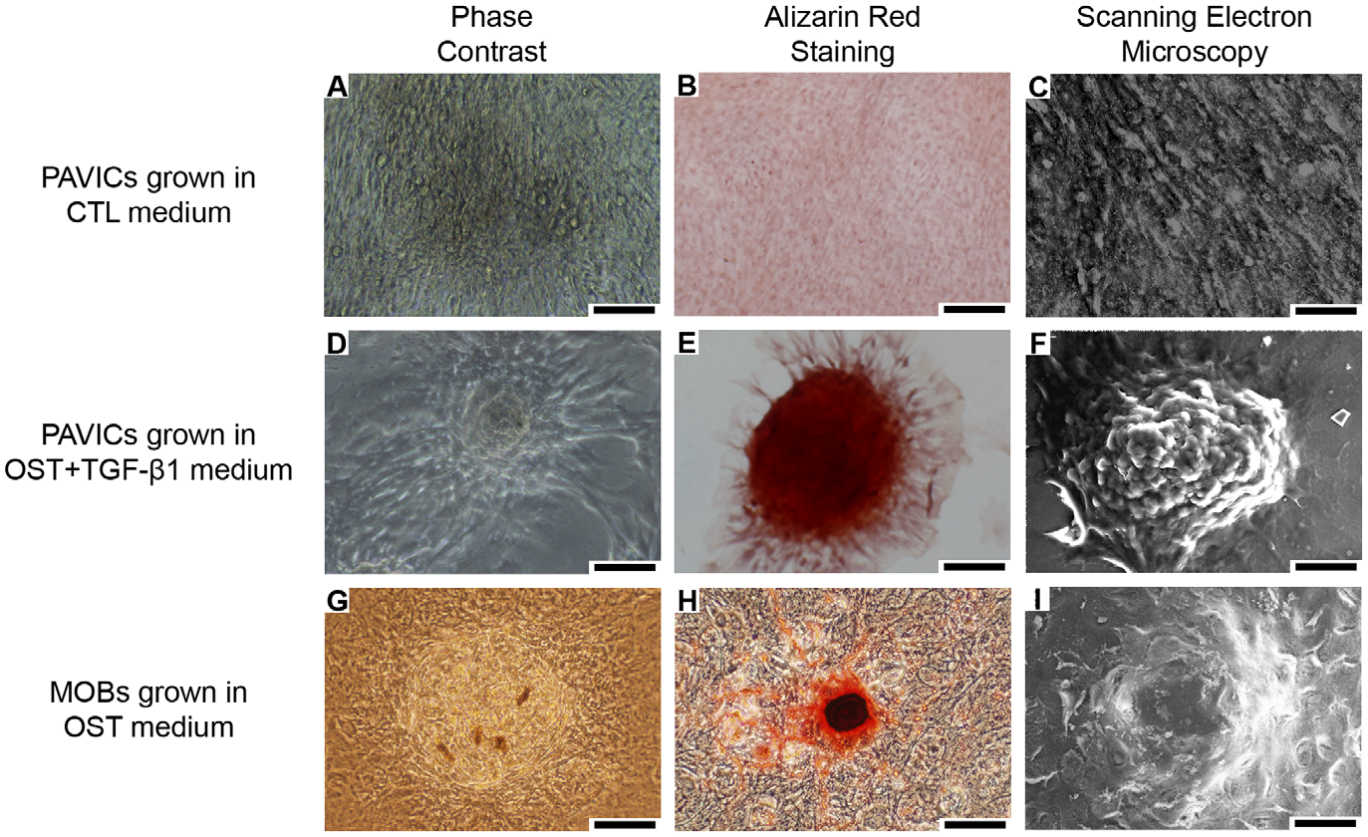
Micrographs showing the morphology and staining of PAVICs and MOBs in culture. **A,D,G -** Phase contrast images of cultured PAVICs grown in CTL medium for 21 days, PAVICs grown in OST+TGF-β1 medium for 21 days, and MOBs grown in OST medium for 21 days respectively (scale = 100 µm). **B,E,H -** Alizarin Red S staining negative for PAVICs grown in CTL medium for 21 days, positive for PAVICs grown in OST+TGF-β1 medium for 21 days, and positive for MOBs grown in OST medium respectively (scale = 100 µm). **C,F,I** - SEM images of cultured PAVICs grown in CTL medium for 21 days, PAVICs grown in OST+TGF-β1 medium for 21 days, and MOBs grown in OST medium for 21 days respectively (scale = 50 µm).

VICs cultured in CTL and OST+TGF-β1 media for 21 days produced distinct, dense nodules approximately 50–200 µm in diameter (Figure 1A and D, respectively) with nodule formation occurring as early as day 4 in culture. Whilst nodules formed in CTL medium were visibly distinguishable from the surrounding monolayer, those formed in OST+TGF-β1 medium were more raised from the culture surface and appeared more compact. Nodules were similar in gross appearance to those formed from MOBs (Figure 1G). Nodules formed from PAVICs grown in OST+TGF-β1 media stained positively for ARS, a calcium stain, after 21 days in culture (Figure 1E). Nodules formed in CTL medium did not stain positively for ARS (Figure 1B), whilst MOBs nodules did (Figure 1G). SEM images demonstrated that nodules formed from PAVICs cultured in OST+TGF-β1 medium produced distinct three-dimensional morphologies (Figure 1F), whereas nodules formed in CTL medium appeared as dense areas of cell growth with a less raised profile from the cell monolayer (Figure 1C). Nodules formed from MOBs had similar three-dimensional morphologies to those observed in the PAVICs cultures grown in OST+TGF-β1 medium (Figure 1I).

### PAVICs Grown in OST+TGF-β1 Medium Show an Increase in type I Collagen Expression

**Figure 2:**
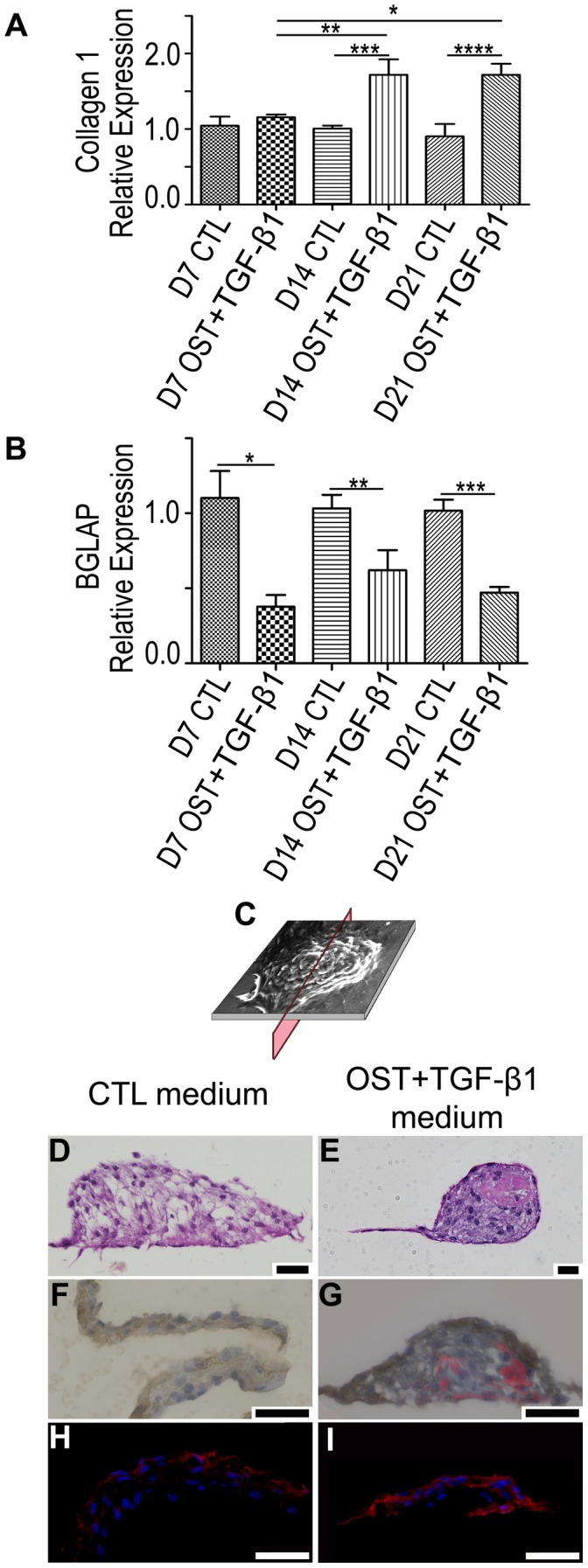
Gene expression and cross sectional staining of cultured PAVICs. **A -** Gene expression of collagen I (* = p<0.05, ** = p<0.0001, *** = p<0.05;**** = p<0.001) and **B -** Osteocalcin (bone gamma-carboxyglutamate protein (BGLAP)) (* = p<0.05; ** = p<0.001; *** = p<0.0001) comparing PAVICs expression at day 7 (D7), day 14 (D14), and day 21 (D21) in both OST+TGF-β1 and CTL media. **C –** Cross sectional plane of cultured nodules used for the D,E,F,G,H, and I. **D,E -** Modified Verhoeff van Gieson stain [purple – cell nuclei, pink – collagen, black-elastin] of a cross section of PAVICs cultured in CTL media for 21 days and PAVICs cultured in OST+TGF-β1 media for 21 days respectively (scale = 50 µm). **F,G -** Peroxidase stain with Sirius red staining [brown - αSMA, red – collagen] for PAVICs grown in CTL medium for 21 days and PAVICs grown in OST+TGF-β1 medium for 21 days respectively (scale = 50 µm) **H,I -** Fluorescence staining [red - αSMA, blue - cell nuclei] of a PAVICs nodule cultured for 21 days in CTL medium and a PAVICs nodule cultured in OST+TGF-β1 medium for 21 days respectively (scale = 50 µm).

PAVICs grown in OST+TGF-β1 media showed a significantly higher expression of collagen type I after 14 and 21 days in culture compared to the expression levels after 7 days (*p*<0.05) and to PAVICs cultured in CTL medium at the same time points (*p*<0.05, Figure 2A). PAVICs grown in CTL medium also showed no changes in type I collagen expression at any of the three time points examined.

*BGLAP* (osteocalcin) was stably expressed in PAVICs grown in OST+TGF- β1 or CTL media, however, its expression was significantly lower (*p*<0.05) in cells cultured in OST+TGF-β1 medium when compared to cells grown in CTL medium after 7, 14 and 21 days, (Figure 2B).

### OST+TGF-β1 Medium Induces αSMA Expression and Collagen Deposition in PAVICs

Modified Verhoeff van Geison staining and sirius red identified collagen in PAVICs nodules formed in OST+TGF-β1 medium. This was in contrast to nodules spontaneously formed in CTL medium where no collagen deposition was visualized in either the monolayer or nodule cross sections. No elastin was observed in PAVICs cultures treated with either CTL or OST+TGF-β1 media. αSMA was present in CTL- and OST+TGF-β1-derived nodules and monolayers, however, was not expressed homogeneously throughout the nodules, but rather only in the outermost layers (Figure 2 G, I).

### TEM of OST+TGF-β1 PAVICs Nodule Cross Sections Show Abundant ECM and no Evidence of Mineralization

**Figure 3:**
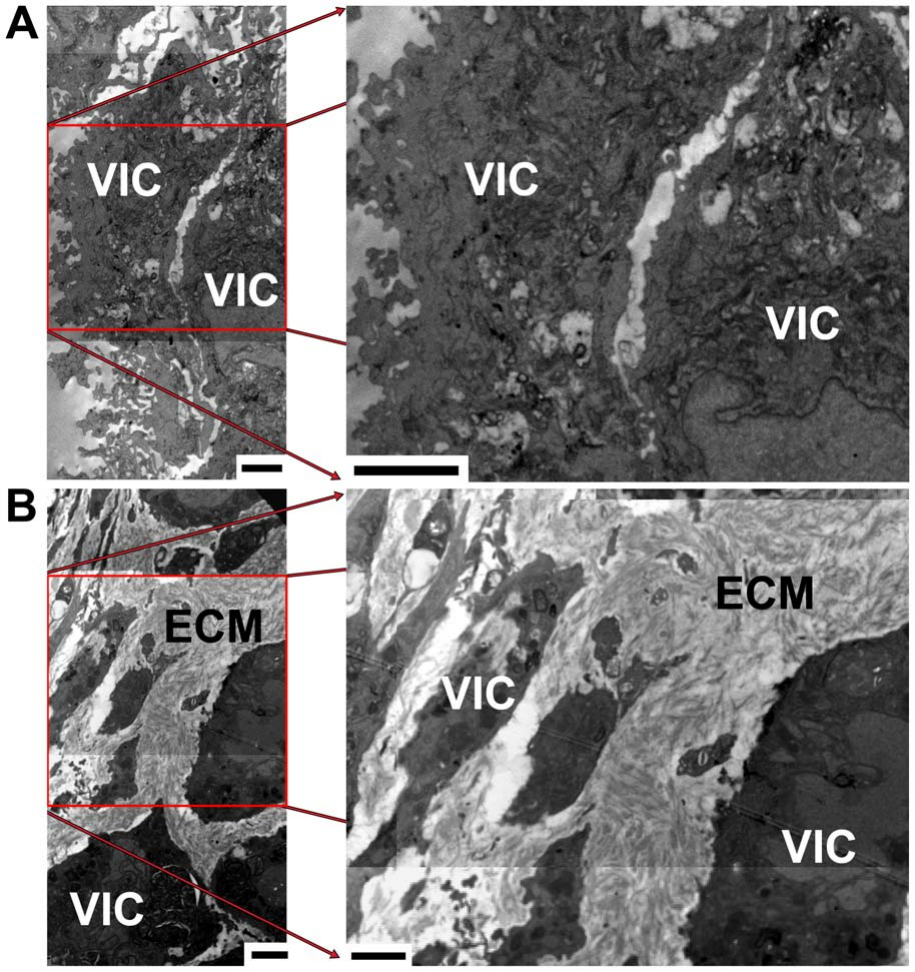
Transmission Electron Micrographs of cultured PAVICs. PAVICs were grown in **A -** CTL medium and **B -** OST+TGF-β1 medium (VIC – valvular interstitial cell, ECM – extracellular matrix) (scale = 2 µm).

TEM images of PAVICs nodule cross sections showed layered cellular aggregates similar to those identified by histology. PAVICs nodules grown in OST+TGF-β1 medium contained a proteinaceous ECM which was apparent throughout the nodules and surrounding cells (Figure 3A). The proteinaceous ECM appeared disorganized and contained fibrous proteins with a banded appearance (approximately 65 nm in periodicity) indicative of mammalian collagen, specifically type I collagen [33]–[35]. PAVICs nodules formed in CTL medium displayed a rough membrane and contained little to no proteinacious ECM between cells (Figure 3B). Electron dense mineral deposits were not evident in any PAVICs cross sections.

### Raman Spectroscopy Exposes High Protein Content in OST+TGF-β1 PAVICs Nodules without any Mineral Presence

**Figure 4:**
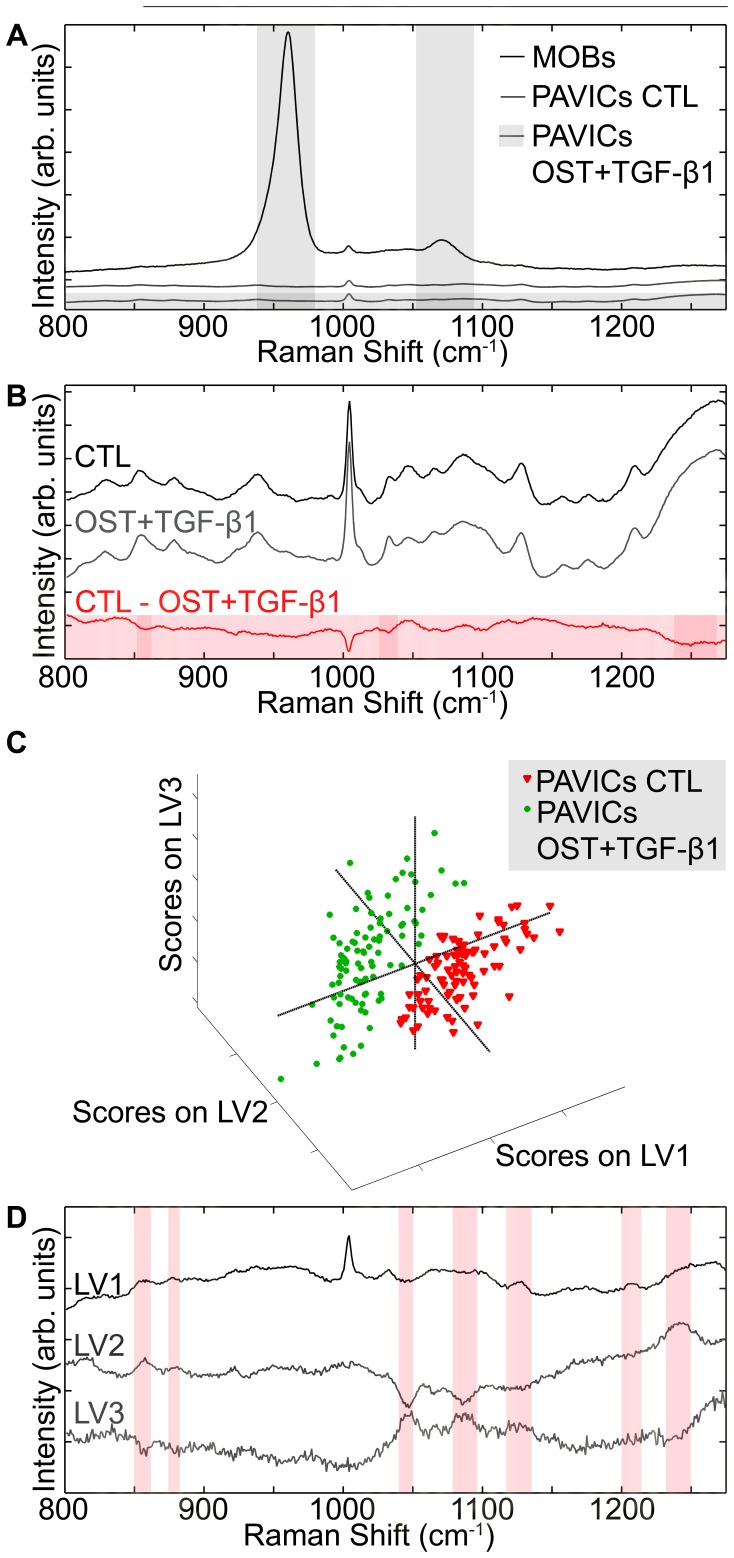
Raman spectroscopy of PAVICs and MOBs in culture compared through univariate and multivariate statistical analysis. **A -** Raman spectra comparing a representative MOBs mineralized nodule spectrum (black), PAVICs grown in CTL media mean spectrum (dark grey), and PAVICs cultured in OST+TGF-β1 medium mean spectrum (shaded box over entire spectrum). Vertical shaded areas highlight the 960 cm^−1^ apatite peak and the 1070 cm^−1^ carbonate peak spectral range. **B -** Higher magnification of the PAVICs mean spectrum grown in CTL (dark grey) and in OST+TGF-β1 medium (light grey). The red spectrum is the difference between the two PAVICs mean spectra. Shaded red bands highlight spectral ranges which discriminate between groups. **C -** Scatter plot showing group separation between PAVICs grown in CTL medium (red triangles) vs. PAVICs grown in OST+TGF-β1 medium (green circles). **D -** Latent variables loadings used in the interval partial least squares discriminant analysis (iPLS-DA) model, bands highlighted are spectral ranges which contributed to the distinction between groups.

Inorganic peaks indicative of mineralization were not present in CTL (day 21), CTL+TGF-β1 (day 14), OST (day 14) or OST+TGF-β1 (day 21) treated PAVIC nodules. In contrast the 960 cm^−1^ and 1070 cm^−1^ mineral peaks identifying the phosphate PO_4_ bonds and type B carbonate substitution CO_3_ bonds, respectively, are clearly seen in the spectrum collected from an MOB nodule (Figure 4A). Raman maps of PAVICs nodules grown in CTL (day 21), CTL+TGF-β1 (day 14), OST (day 14) or OST+TGF-β1 (day 21) demonstrated a clear absence of mineral throughout the entire area of the nodules imaged (Figure S1). Mineral bands were present in spectra collected from calcified human aortic valve tissue as seen in the representative mean spectrum (Figure S2).

The difference spectrum between CTL and OST+TGF-β1 (red spectrum in Figure 4B) reveals that protein bonds contributed the majority of the difference between the groups. Specifically, bands at 855, 874, 1043 and 1245–1270 cm^−1^ corresponding to C-C stretch proline, C-C stretch hydroxyproline, proline and Amide III, respectively, are notable. The hydroxyproline and two proline peaks identified in these spectra are specifically Raman collagen assignments [36] confirming a collagen presence within the PAVICs grown in OST+TGF-β1 medium.

The iPLS-DA model derived from spectra collected from PAVICs nodules grown in CTL or OST+TGF-β1 media for 21 days identified three latent variables which differentiate between the two groups with an equal sensitivity and specificity of 0.979. A clear division emerged between PAVICs grown in CTL and OST+TGF-β1 media, as is evident in the scatter plot which identifies the loadings of each collected spectra (Figure 4C). The latent variables used in the iPLS-DA model are also shown in Figure 4C. These variable loadings show spectral regions which varied between the experimental groups. The latent variables are shown with highlighted bands indicating spectral regions which highly contributed to the differentiation between experimental groups. Specifically, bands at 855, 874, 1032, 1206, 1247 and 1665 cm^−1^ are collagen assignment groups. Table 1 lists all bands (mineral and organic) highlighted in both the univariate and multivariate Raman analysis with their corresponding assignments.

**Table 1:** Raman bands contributing to the distinction between PAVICs grown in OST+TGF-β1 media, CTL media and MOBs mineralised nodules.

Peak position (cm^−1^)	Major Assignments
**855**	C-C stretch, proline*
**874**	C-C stretch, hydroxyproline*
***960***	*Apatite - PO_4_^3^* ^−^ *v_1_ symmetric stretch*
**1003**	Phenylalanine ring breathing mode
**1032**	Proline *
**1043**	Proline*
***1070***	*Carbonate - substituted CO_3_^2^* ^−^ *v_1_ in-plane vibrations*
**1086**	C–N stretching mode of proteins (and lipid mode tolesser degree)
**1128**	C-N
**1206**	Hydroxyproline, tyrosine*
**1247–1270**	Protein amide III band
**1,595–1,720**	Amide I (C = O stretching mode of proteins, α-helixconformation)/C = C lipid stretch

Inorganic peaks are italicized. * indicates collagen assignments.

## Discussion

The mechanism that drives valve calcification has been likened to that of bone formation and/or a dystrophic process which includes the deposition of hydroxyapatite mineral [4], [21], [24], [37]; nevertheless the process which mediates the formation of calcified lesions on aortic valve cusps remains uncertain. Investigators have speculated that valve calcification involves a transdifferentiation of VICs into osteoblasts, which then mediate bone-like mineral formation [6], [37], [38]. The implication of such an osteoblast-like mineralization process in the aortic valve has prompted the development of *in vitro* models to examine the disease process. Easily cultured and fast-growing PAVICs are often used as a simplified model for aortic valve calcification [18], however, their efficacy in representing the disease has yet to be established. This study aimed to characterize the ‘calcified’ OST+TGF-β1 PAVICs nodule composition, compare them to those created by a confirmed mineralizing culture model (MOBs), and report any calcium phosphate deposition within the PAVICs nodules.

We observed that PAVICs grown in OST+TGF-β1 medium formed nodular structures that stained positively for ARS, as has been previously described [17], [39]. Such nodules were notable for their distinct three-dimensional morphologies that are reminiscent of nodules formed from MOBs, which also stained positively for ARS. Nevertheless, when we examined the ultrastructure of such nodules by TEM, no electron-dense mineral deposits were observed, as were readily identifiable in our previously published report of nodules formed from MOBs [40]. Furthermore, Raman spectroscopy measurements clearly showed an absence of mineralization in the OST+TGF-β1 PAVICs, whilst MOBs nodules demonstrated distinct peaks, indicative of phosphate and type-B carbonate-substituted mineral. These results suggest that ‘calcified’ nodules formed from PAVICs under the conditions examined here do not form mineral deposits and that ARS staining is a poor method to identify mineral deposits in such cultures. Notwithstanding, these data do not preclude the possibility of osteogenic transdifferentiation of PAVICs or exclude the chance that they could form mineral under different conditions.

A number of studies have suggested that PAVICs and human aortic VICs may differentiate to osteoblast-like cells during calcified valve disease progression [41]–[43]. A study by Chen *et al*. demonstrated that both mesenchymal and osteogenic progenitor cells exist within the primary PAVICs mixed cell population. Their results further exposed that PAVICs have the ability to transdifferentiate into myofibrogenic, adipogenic, osteogenic and chodrogenic lineages *in vitro* and thus potentially *in vivo* [41]. To probe PAVICs nodules’ potential for osteoblastic differentiation when grown in OST+TGF-β1 medium, we examined expression of two genes: type I collagen (*COL1A1*) and osteocalcin *(BGLAP)*. It has been reported that calcified human aortic valve interstitial cells have an increase in osteocalcin RNA expression (a late marker for bone differentiation) [16]. Whilst we noted an up regulation of type I collagen in OST+TGF-β1 grown PAVICs, *BGLAP* expression remained stable in PAVICs grown in OST+TGF-β1 and this level of expression was significantly lower than expression levels in CTL PAVICs at the same time points. This suggests that PAVICs grown in OST+TGF-β1 for up to 21 days were not differentiating into osteoblasts. A previous study demonstrated PAVICs grown in mineralization medium for up to eight days did not display the same level of increased alkaline phosphatase (an early mineralization marker) as osteoblasts in culture [44]. The lack of osteoblastic differentiation in this study may be attributed to a wide range of factors including their growth on stiff tissue culture plastic/glass substrates [43] and/or TGF-β1 supplementation.

In this study the OST media was supplemented with TGF-β1 due to its physiological importance in tissue calcification. Studies have shown qualitatively higher levels of TGF-β1 in the ECM that co-localized with areas of calcification in diseased human aortic valves [17], [19]. Additionally, the inflammatory response has been implicated as an important contributing factor in disease onset, which suggests that a local availability of TGF-β1 [6], [45] may increase during the initial stages of disease progression. Nevertheless, the connection between TGF-β1 use in this *in vitro* system and disease progression is still unclear. Osman *et al*. showed that supplementation of human VIC cultures with members of the TGF-β family (including TGF-β1) prompted the cells to adopt a more osteoblast-like phenotype by inducing the secretion of proinflammatory cytokines which may play an important role in pathological valvular calcification [42]. Our results here show that PAVICs grown in OST+TGF-β1 medium do not show evidence of osteoblastic transdifferentiation and thus these ‘calcified nodules’ have yet to demonstrate their relationship to calcified aortic valve disease progression. The absence of mineral within CTL+TGF-β1 and OST medium PAVICs nodules suggests the lack of mineralization is not due to osteogenic supplementation or the additional TGF-β1 supplementation.

TEM and histological staining demonstrated that PAVICs nodules grown in OST+TGF-β1 were marked by an abundant proteinaceous ECM, which contained collagen, but without a specific arrangement or orientation. Collagen production is mediated by VICs *in vivo* as part of normal valve maintenance, however, disruption of this process has also been associated with calcified aortic valve disease progression [46]. Valvular fibrosis and over-activated VICs have been implicated in the early stages of calcified valve pathobiology [46], including a recent suggestion of calcified aortic valve stenosis being more appropriately viewed as a fibrocalcific disease [47]. Our current study confirms TGF-β1 supplementation likely promotes and/or maintains an activated myofibroblastic phenotype in PAVICs and production of ECM *in vitro*. The relationship, if one exists, between *in vitro* PAVIC-mediated ECM production and the fibrotic stage of aortic valve calcification, however, has yet to be established.

PAVICs-mediated production of fibrous ECM was further explored using Raman spectroscopy. Like the histological analyses, Raman spectroscopy further identified the abundant proteinaceous content of PAVICs nodules cultured in OST+TGF-β1 medium. Our PLS-DA model successfully distinguished between the two experimental PAVICs systems based on the ECM produced by PAVICs grown in OST+TGF-β1 medium. The model also clearly indicated the collagen content within the nodules was a heavy contributor to the model variables, and thus collagen is a distinguishing element between the groups. Taken together, these results suggest Raman spectroscopy may be an effective means to successfully and non-invasively monitor ECM production in live PAVIC systems *in vitro*.

Cross sections of OST+TGF-β1 PAVICs nodules showed positive expression of αSMA, as did monolayers of PAVICs grown in CTL medium, implicating a myofibroblastic phenotype [48]. Our results show that the cells at the centre of these nodules are not αSMA positive and thus may have a different phenotype or be undergoing apoptosis, as has been previously suggested [39]. TGF-β1 has been suggested to promote myofibroblastic expression particularly when incorporated on stiff substrates [43] and calcification via an apoptosis pathway [17], [46], thus suggesting a non-osteoblastic state [17]. The results presented in this study show that PAVICs cultured in OST+TGF-β1 medium for 21 days, a relatively late time point for in vitro culture [44], do not further transdifferentiate from the activated myofibroblastic phenotype into osteoblast-like cells or contain calcium phosphate within the ‘calcified nodules’. It remains uncertain as to whether VICs must pass through an intermediate stage of activated VICs to become osteoblast-like VICs [46], [47]. Further investigations are needed to establish if the OST+TGF-β1 PAVICs model has any relationship to early (preosteoblastic) stages of calcified aortic valve disease.

The inhibition of nodule formation in cultured VICs has been explored through the addition of statins, pravastatin [49], nitric oxide donors, as well as other cell permeate superoxide scavengers [20]. The response and transformations of VICs grown *in vitro* to various treatments raise interesting questions regarding the relationship between these cells and the complex *in vivo* environment. As investigations into the pathobiology of aortic valve calcification progress, characterization of both systems using a variety of techniques offers promise of bridging this gap.

This study combines gold standard biological techniques as well as advanced material characterization techniques including rapid, non-invasive Raman spectroscopy. The results show PAVICs grown in OST+TGF-β1 media for up to 21 days express an activated myofibroblastic phenotype and produce a predominantly collagen ECM, however, demonstrate no evidence of further transdifferentiation into an osteoblastic phenotype and/or calcium phosphate deposition. Additionally these PAVICs nodules did not contain any calcium phosphate materials as seen in human aortic valve calcification. We have thus established a clear limitation of cultured PAVICs grown in CTL and OST+TGF-β1 media as they do not appear to transdifferentiate into osteoblastic-like cells nor form mineral deposits indicative of calcified aortic valve disease [50]. This study also provides further information on the collagen-rich ECM produced in PAVICs nodules grown in OST+TGF-β1 medium and the heterogeneous nature of these nodules. This characterisation of *in vitro* PAVICs systems is critical in further understanding PAVICs behavior in culture and for comparison to aortic valve calcification.

## Supporting Information

Figure S1 **Raman maps of PAVICs nodules grown in vitro.** **A -** White light micrograph of PAVICs grown in CTL+ TGF-β1 (scale = 20 µm). **B -** Overlay of a Raman map of the phenylalanine peak onto the white light micrograph in **A**, showing the cellular presence as seen within the collected Raman spectra (scale = 20 µm). **C** **-** Overlay of the apatite peak Raman map onto the white light micrograph in A showing no mineral was detected in any of the mapped area (scale = 20 µm). **D -** Representative Raman spectra from Raman maps of PAVICs nodules grown CTL medium for 21 days, OST+TGF-β1 medium for 21 days, OST medium for 14 days and CTL+TGF-β1 for 14 days. The phenylalanine (1003 cm^−1^), amide III (1214–1270 cm^−1^), and CH_2_ bending (1445 cm^−1^) peaks clearly identified cellular areas within the Raman maps. The absence of mineral peak, including the 960 cm^−1^ apatite and 1070 cm^−1^ carbonate peak show that there is no mineral presence within these nodules, and mineral associated peaks were not seen in any spectra collected from the PAVICs.
(TIF)

Figure S2 **Raman spectra collected from calcified human aortic valves.** Mean Raman spectrum of 128 spectra collected from independent locations within calcified human aortic valve tissue (isolated from 4 separate donors). The grey bands highlight the mineral peaks present within the collected spectra at 960 cm^−1^ (apatite) and 1070 cm^−1^ (carbonate peak).
(TIF)

Methods S1 (DOC)
