# Supplementary figures and images for: Characterization of Porcine Aortic Valvular Interstitial Cell ‘Calcified’ Nodules
Source: PLoS One. 2012 Oct 26;7(10):e48154. doi: 10.1371/journal.pone.0048154 (PMC3482191; doi:10.1371/journal.pone.0048154)

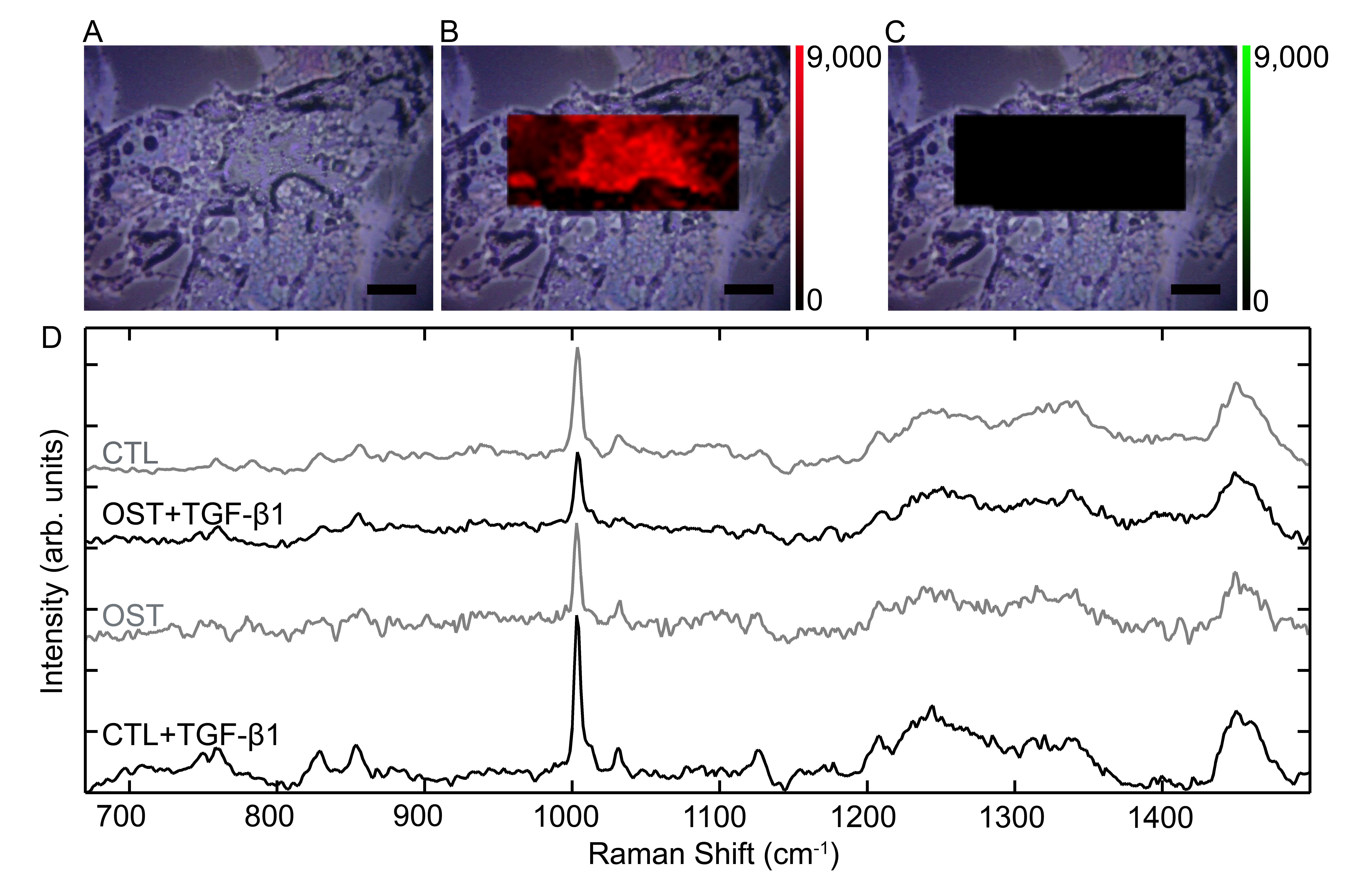

Supplement: Figure S1 — Raman maps of PAVICs nodules grown in vitro. A - White light micrograph of PAVICs grown in CTL+ TGF-β1 (scale = 20 µm). B - Overlay of a Raman map of the phenylalanine peak onto the white light micrograph in A, showing the cellular presence as seen within the collected Raman spectra (scale = 20 µm). C - Overlay of the apatite peak Raman map onto the white light micrograph in A showing no mineral was detected in any of the mapped area (scale = 20 µm). D - Representative Raman spectra from Raman maps of PAVICs nodules grown CTL medium for 21 days, OST+TGF-β1 medium for 21 days, OST medium for 14 days and CTL+TGF-β1 for 14 days. The phenylalanine (1003 cm−1), amide III (1214–1270 cm−1), and CH2 bending (1445 cm−1) peaks clearly identified cellular areas within the Raman maps. The absence of mineral peak, including the 960 cm−1 apatite and 1070 cm−1 carbonate peak show that there is no mineral presence within these nodules, and mineral associated peaks were not seen in any spectra collected from the PAVICs. (TIF) [file pone.0048154.s001.tif]

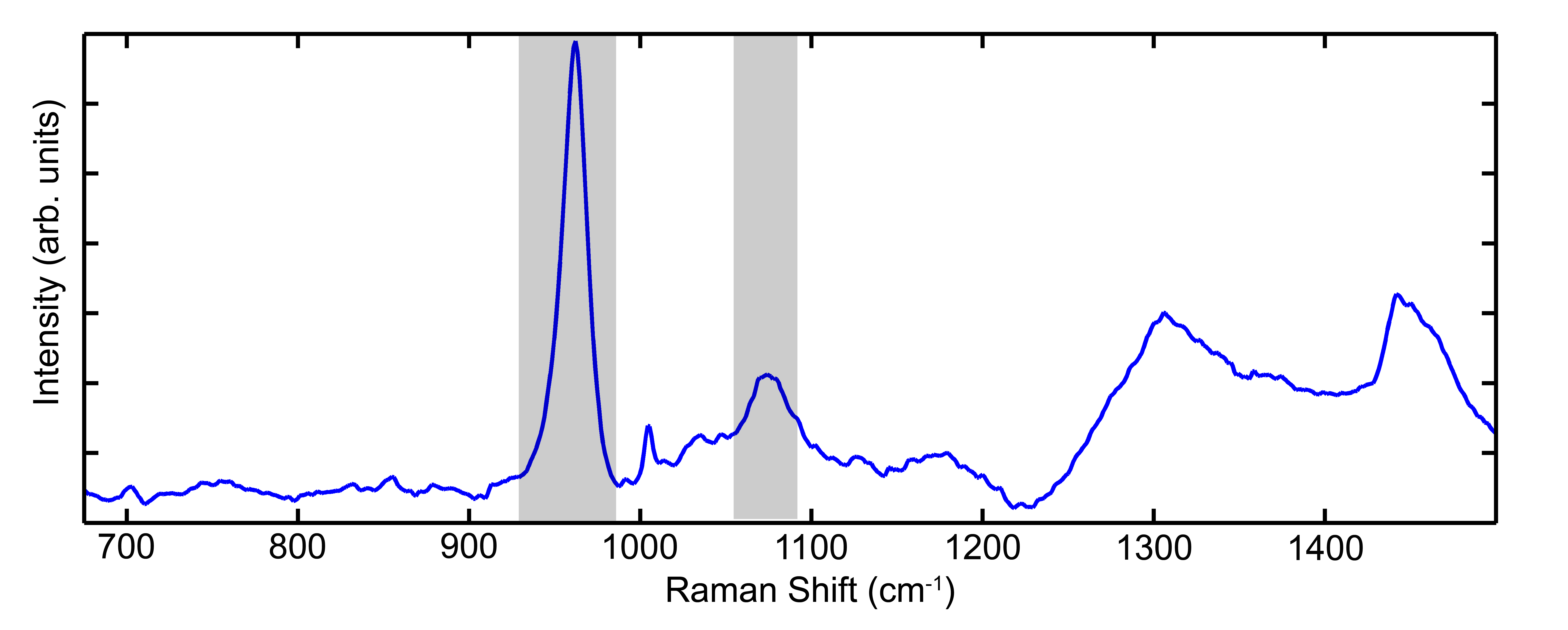

Supplement: Figure S2 — Raman spectra collected from calcified human aortic valves. Mean Raman spectrum of 128 spectra collected from independent locations within calcified human aortic valve tissue (isolated from 4 separate donors). The grey bands highlight the mineral peaks present within the collected spectra at 960 cm−1 (apatite) and 1070 cm−1 (carbonate peak). (TIF) [file pone.0048154.s002.tif]
