# Supplementary material for: Characterization of Porcine Aortic Valvular Interstitial Cell ‘Calcified’ Nodules
Source: PLoS One. 2012 Oct 26;7(10):e48154. doi: 10.1371/journal.pone.0048154 (PMC3482191; doi:10.1371/journal.pone.0048154)
Supplement: Methods S1 — (DOC) [file pone.0048154.s003.doc]

**Supplementary Methods**

**Calcified human aortic valve isolation**

Calcified human aortic valves were obtained from an Old Brompton Hospital, London, U.K. A total of four patients were included in this investigation at ages 51, 55, 63 and 81 years old. After collection each cusp was isolated, fixed in 4% (w/v) formaldehyde (FA) for 48 hours at 4°C and then rinsed in phosphate buffered saline (PBS). The cusps were then dehydrated in a graded ethanol series and calcified tissue was isolated for testing.

**Raman Spectroscopy**

Human tissue spectra were collected with a 785nm laser, using a Renishaw InVia spectrometer connected to a Leica microscope as previously described . Briefly, spectra were collected from dry isolated human aortic valve cusp calcified tissue at room temperature. Spectra were collected over 3 accumulations of 5 second scans covering the Raman shifts range of 618-1728cm^-1^.

Raman spectra were processed and analysed with software developed internally for use with Matlab (The Mathworks) environment and with the multivariate statistical analysis PLS toolbox (Eigenvector Research). Raman spectra were intensity-corrected for instrument response, background subtracted using the Modpoly algorithm (5th order polynomial, 1000 iterations) and lightly smoothed using a 5-point Savitsky-Golay filter (2nd order polynomial) as previously described [29].
